# Supplementary material for: Electronic heat flow and thermal shot noise in quantum circuits
Source: Nat Commun. 2019 Dec 10;10:5638. doi: 10.1038/s41467-019-13566-8 (PMC6904624; doi:10.1038/s41467-019-13566-8)
Supplement: Supplementary file 1 — Supplementary Information [file 41467_2019_13566_MOESM1_ESM.pdf]

Supplementary Figures for
‘Electronic heat flow and thermal shot noise in quantum circuits’

E. Sivre,[1] H. Duprez,[1] A. Anthore,[1,2] A. Aassime,[1] F.D. Parmentier,[1]
A. Cavanna,[1] A. Ouerghi,[1] U. Gennser,[1] and F. Pierre[1]

[1] *Université Paris-Saclay, CNRS, Centre de Nanosciences et de Nanotechnologies (C2N), 91120 Palaiseau, France*
[2] *Université de Paris, C2N, 91120 Palaiseau, France*

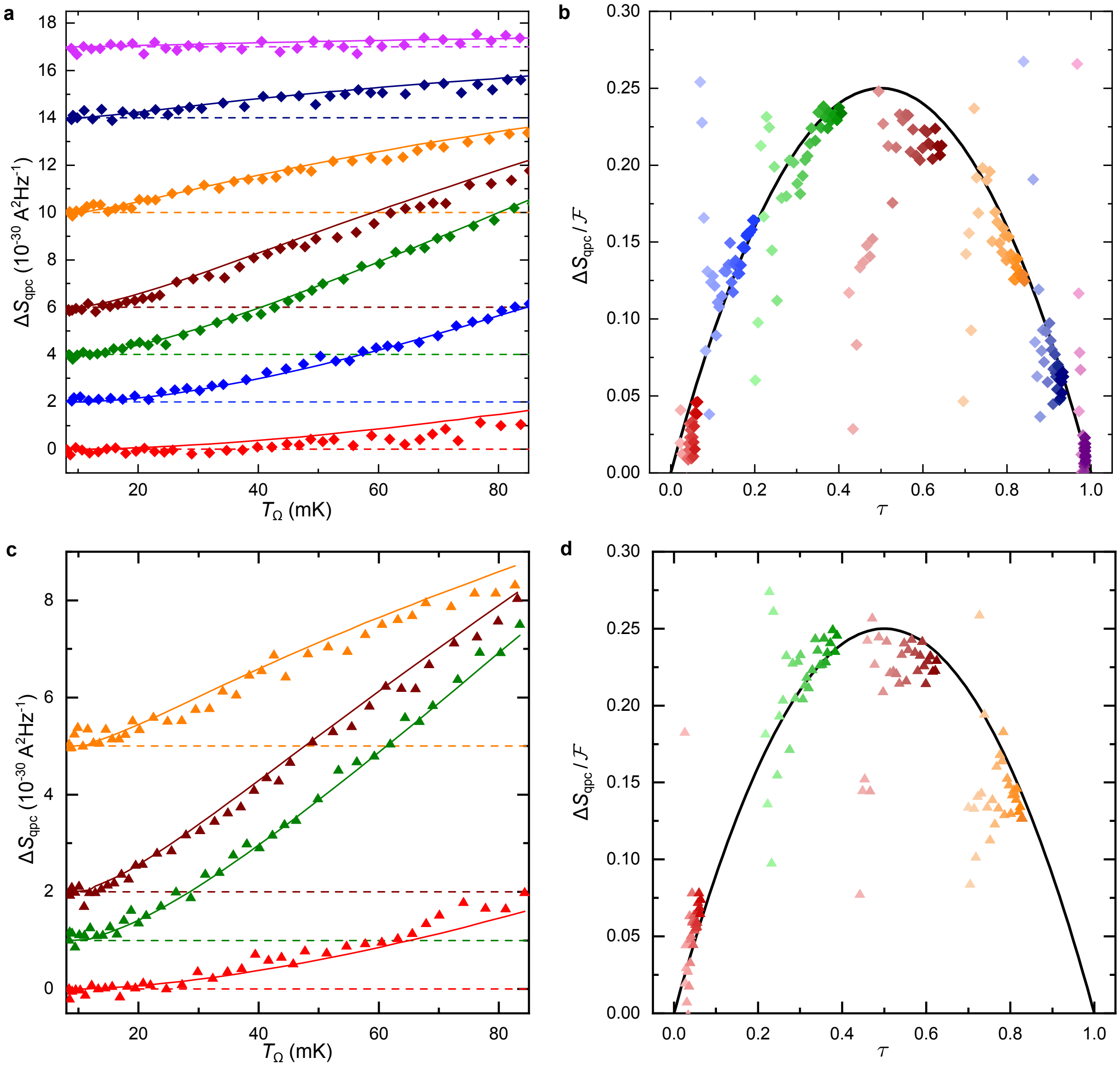

Supplementary Figure 1. Thermal shot noise in additional circuit configurations at base temperature $T \simeq 8$ mK. The thermal shot noise data shown in Fig. 2 represent measurements performed with the circuit tuned at $N = 2$. Here, we display the thermal shot noise and $\Delta S_{\rm qpc}/\mathcal{F}$ measured with $N = 3$ in panels (a), (b) and $N = 4$ in panels (c), (d).

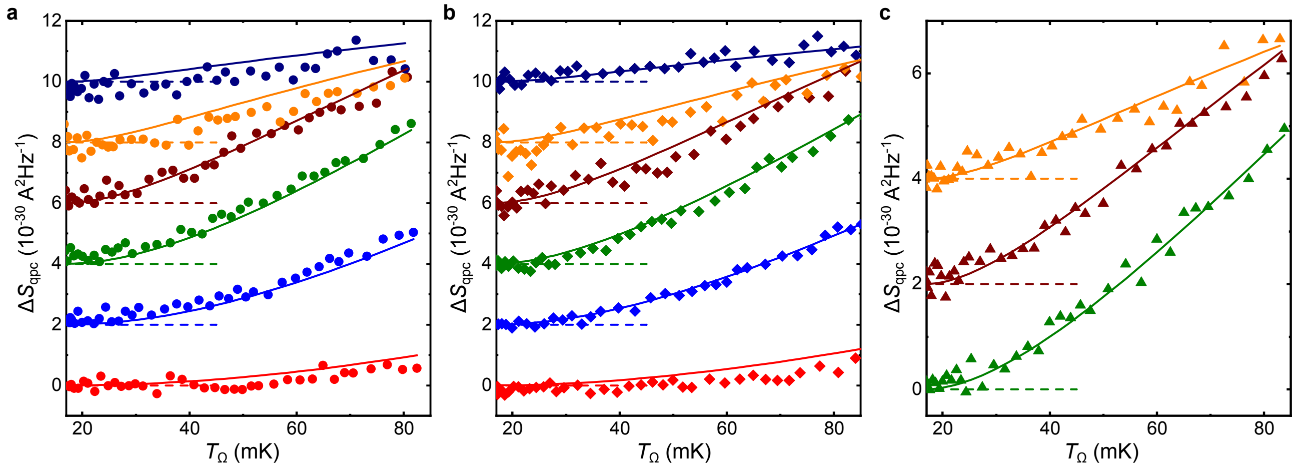

Supplementary Figure 2. Thermal shot noise at $T \simeq 16\,\mathrm{mK}$. The figure displays similar thermal shot noise data as those shown in Fig. 2a and in Supplementary Fig. 1a,c, but here measured at a larger base temperature $T \simeq 16\,\mathrm{mK}$. The panels (a), (b) and (c) display as symbols the experimental thermal shot noise obtained, respectively, in the circuit configurations $N = 2$, 3 and 4. Continuous lines are the quantitative predictions of Eq. 1, without adjustable parameter. Different colors represent different QPC settings (same setting as corresponding $T \simeq 8\,\mathrm{mK}$ data, shown with the same color). Plots are offset for clarity with the offsets displayed as horizontal dashed lines.

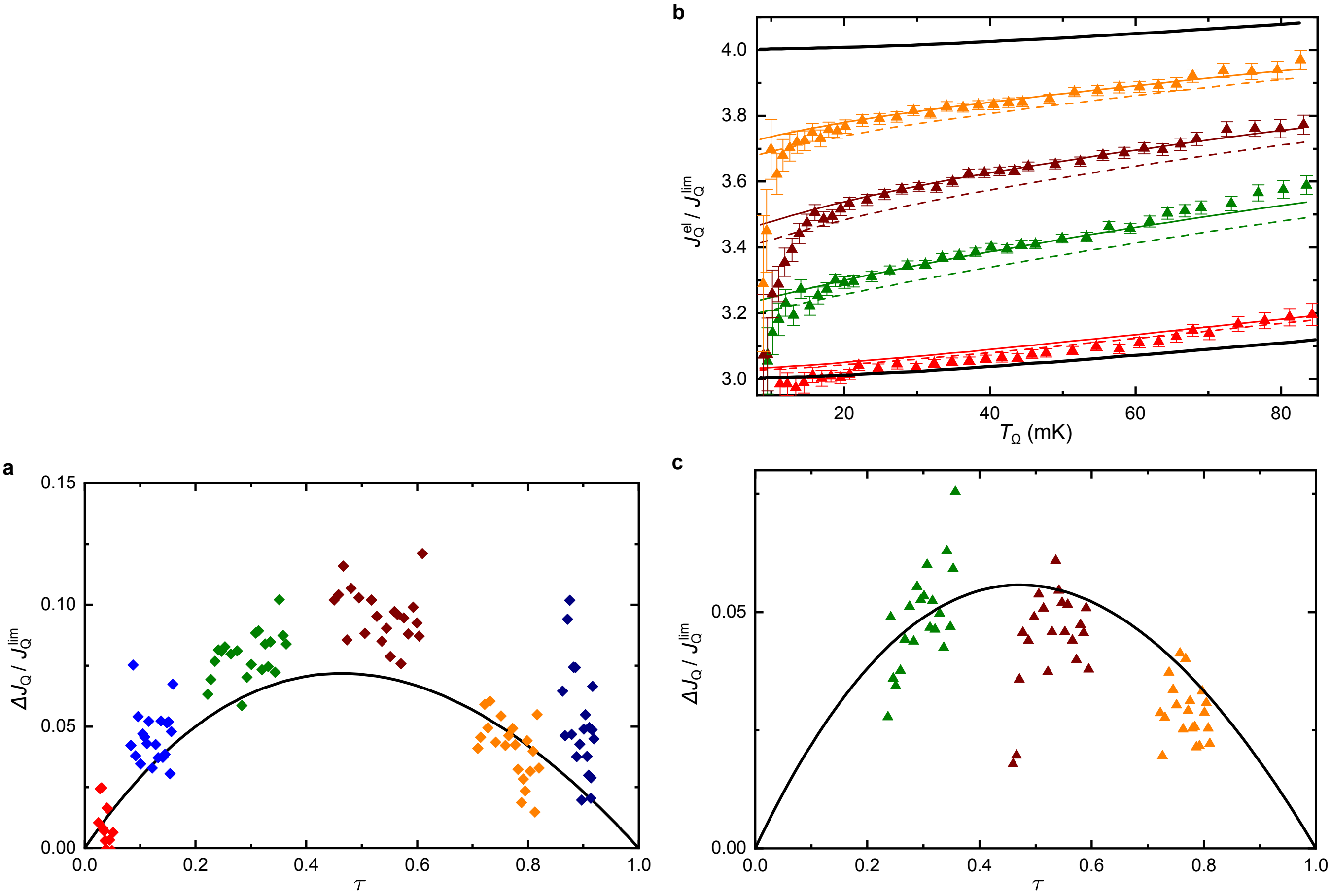

Supplementary Figure 3. Electronic heat flow in additional circuit configurations at $T \simeq 8\,\mathrm{mK}$. This Supplementary Figure complements Fig. 3 by showing $\Delta J_Q^{\mathrm{el}}/J_Q^{\mathrm{lim}}$ for $N = 3$ (a) and $N = 4$ (c), and by showing $J_Q^{\mathrm{el}}/J_Q^{\mathrm{lim}}$ for $N = 4$ (b). Error bars in (b) represent the standard statistical error.

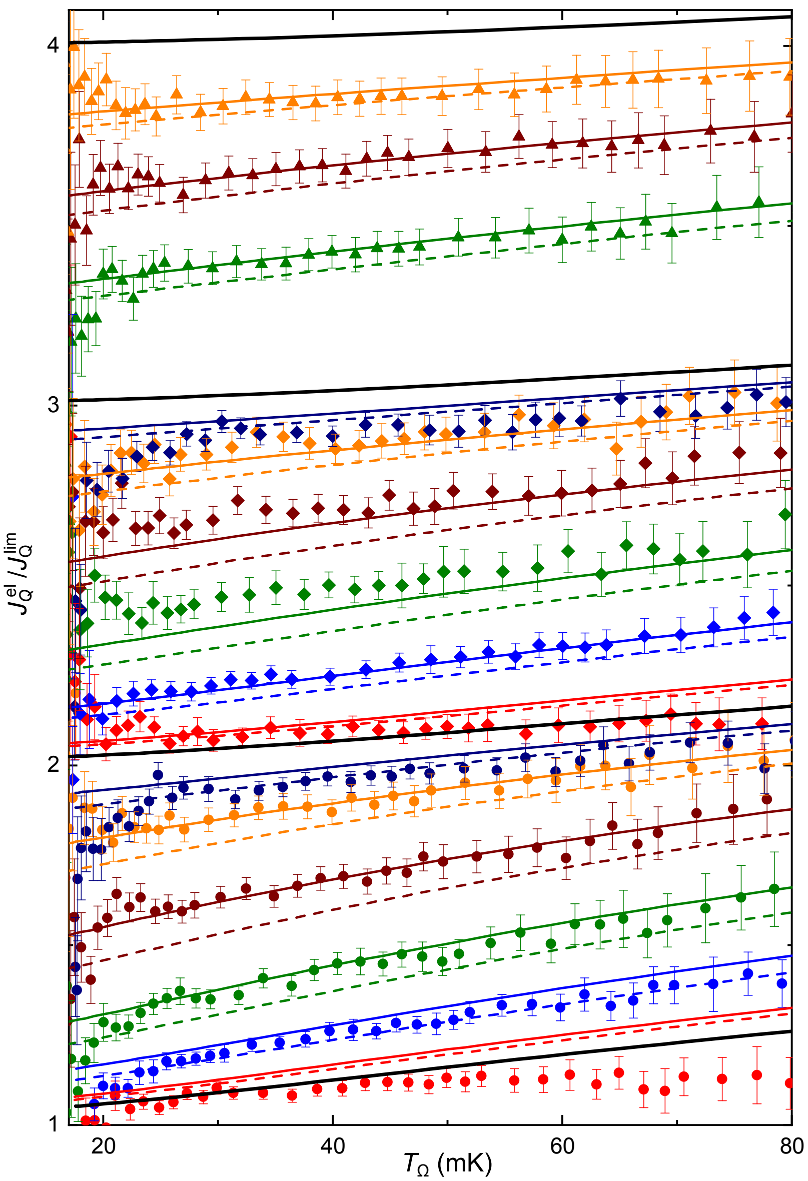

Supplementary Figure 4. Electronic heat flow at $T \simeq 16\,\mathrm{mK}$ for different circuit settings ($N \in \{2,3,4\}$) as a function of the temperature T_Ω of the electrons in the island (similar to the data in Fig. 3a and Supplementary Fig. 3b but at $T \simeq 16\,\mathrm{mK}$). The symbols represent experimental measurements with error bars corresponding to the standard statistical error. The continuous lines show the theoretical predictions of Eq. 23. The dashed lines are linear interpolations between the two nearest ballistic predictions (black continuous lines) weighted by the simultaneously measured $\tau(T_\Omega)$.
